# 27416 DNA Methylation Age Acceleration and Depressive Symptoms in African American Women with Cardiometabolic Conditions

**DOI:** 10.1017/cts.2021.668

**Published:** 2021-03-30

**Authors:** Nicole Perez, Allison Vorderstrasse, Gary Yu, Jacquelyn Taylor

**Affiliations:** 1 New York University; 2 University of Massachusetts at Amherst; 3 Columbia University

## Abstract

ABSTRACT IMPACT: This study deepens knowledge with respect to the associations between depression, cardiometabolic conditions, and accelerated aging with a clinically accessible marker in a population with disproportionate risk for comorbidity. OBJECTIVES/GOALS: The aim of this secondary analysis is to examine associations between DNA methylation age acceleration (DNAm AA) and depressive symptoms in African American women (AAW) considering the presence of cardiometabolic conditions (CMCs) including hypertension, diabetes, obesity. METHODS/STUDY POPULATION: Genomic and longitudinal clinical data (collected 2015-2020) from the Intergenerational Impact of Genetic and Psychosocial Factors on Blood Pressure Study (InterGEN) cohort (n=227) were utilized for this analysis. DNA methylation age (estimated by the Horvath method) incorporates DNA methylation status at 353 CpG sites. DNAm AA is the residual of DNA methylation age regressed on chronological age in a linear model. Spearman’s correlations and linear regression examine the relationship between DNAm AA and depressive symptoms (Beck Depression Inventory) and cardiometabolic status. The potential association and impact of SES, trauma, substance use, and stress were also considered. RESULTS/ANTICIPATED RESULTS: Contrary to our hypothesis, DNAm AA did not associate with the severity of depressive symptoms. Correlation between DNAm AA and affective symptom subscore (BDI) approached significance (p = 0.06). We observed significant correlations between DNAm AA and specific depressive symptoms including participants’ reported disappointment, disgust, or hatred toward themselves (p < 0.05), difficulty with making decisions (p < 0.05), and worry about their physical health (p < 0.05). DNAm AA was also significantly correlated with BMI (p > 0.001). Significant relationships were not evident in the subsequent regression analysis examining potential relationships between DNAm AA and depression. To our knowledge, this is the first study to examine associations between DNAm AA and depressive symptoms in AAW. DISCUSSION/SIGNIFICANCE OF FINDINGS: Depression limits life quality and quantity and is highly comorbid in CMCs. AAW have a high risk of comorbidity. This study deepens knowledge with respect to the associations between depression, CMCs, and aging with a clinically accessible marker in a population with disproportionate risk.

